# Development of a Wireless Health Monitoring System for Measuring Core Body Temperature from the Back of the Body

**DOI:** 10.1155/2019/8936121

**Published:** 2019-02-17

**Authors:** Qun Wei, Hee-Joon Park, Jyung Hyun Lee

**Affiliations:** ^1^Department of Biomedical Engineering, School of Medicine, Keimyung University, Daegu, Republic of Korea; ^2^Department of Biomedical Engineering, School of Medicine, Kyungpook National University, Kyungpook National University Hospital, Daegu, Republic of Korea

## Abstract

In this paper, a user-friendly and low-cost wireless health monitoring system that measures skin temperature from the back of the body for monitoring the core body temperature is proposed. To measure skin temperature accurately, a semiconductor-based microtemperature sensor with a maximum accuracy of ±0.3°C was chosen and controlled by a high-performance/low-power consumption Acorn-Reduced Instruction Set Computing Machine (ARM) architecture microcontroller to build the temperature measuring device. Relying on a 2.4 GHz multichannel Gaussian frequency shift keying (GFSK) RF communication technology, up to 100 proposed temperature measuring devices can transmit the data to one receiver at the same time. The shell of the proposed wireless temperature-measuring device was manufactured via a 3D printer, and the device was assembled to conduct the performance tests and *in vivo* experiments. The performance test was conducted with a K-type temperature sensor in a temperature chamber to observe temperature measurement performance. The results showed an error value between two devices was less than 0.1°C from 25 to 40°C. For the *in vivo* experiments, the device was attached on the back of 10 younger male subjects to measure skin temperature to investigate the relationship with ear temperature. According to the experimental results, an algorithm based on the curve-fitting method was implemented in the proposed device to estimate the core body temperature by the measured skin temperature value. The algorithm was established as a linear model and set as a quadratic formula with an interpolant and with each coefficient for the equation set with 95% confidence bounds. For evaluating the goodness of fit, the sum of squares due to error (SSE), *R*-square, adjusted *R*-square, and root mean square error (RMSE) values were 33.0874, 0.0212, 0.0117, and 0.3998, respectively. As the experimental results have shown, the mean value for an error between ear temperature and estimated core body temperature is about ±0.19°C, and the mean bias is 0.05 ± 0.14°C when the subjects are in steady status.

## 1. Introduction

Health monitoring has always been an important topic in biomedical-engineering research. Body temperature is one of the important numerical values to indicate human health status. The normal body temperature range is typically stated as 36.5 to 37.5°C [1]. The individual body temperature depends on age, exertion, infection, sex, and the place of the body at which the measurement is made [2]. Rectal measurement, oral measurement, and axillary measurement are the well-known methods for human body temperature measurements [3]. However, each method has disadvantages when performing the measurements. The thermometers can break if bitten when doing oral measurement, the rectum could be injured when doing rectal measurements, and the thermometer may need to be left in a place for a long time in order to obtain an accurate measurement. Therefore, the ear thermometer, which measures the temperature of the eardrum, and forehead thermometer, placed on the forehead of the subject to measure the body temperature, were developed. Both methods use infrared sensors to measure temperature, which is different from the mercurial thermometers and standard platinum resistance thermometers used in oral, rectal, and axillary measurements. The infrared thermometer is good for surface temperature measurement and is compact, lightweight, and easy to use. However, the environment needs to be clean, be without dust, and has high humidity. Also, the sensor is expensive, which will raise costs [4, 5].

Recently, because electronic engineering technology is developing rapidly, studies using various electrical devices focused on measuring skin properties objectively, such as measuring and analysis of skin electrical impedance and observing the effects of current, ionic strength, and temperature on the electrical properties of skin [6–11]. However, none of these researches have focused on skin temperature measurement; also, no studies have been conducted to find a relationship with core body temperature. Meanwhile, many medical researchers try to find the relationship between skin temperature and core body temperature for developing a new approach to measure core body temperature by noninvasive methods [12–14]. Researches such as Niedermann et al. have developed an algorithm to predict the core body temperature using the skin temperature measured from the chest. However, the study only used highly professional equipment that is not suitable for longer-term continuous monitoring of subjects in natural habitats or daily environment, so the limited resource is not suitable to develop a complete algorithm to predict the core body temperature.

Lately, Woo et al. proposed a patch-type device that attaches to the skin over the clavicle for measuring skin temperature and humidity and thus to predict the body temperature [15]. The researchers studied the relationship between perspiration rate and skin temperature and used the data to estimate the body temperature. However, the position for affixing the device was not suitable for long-term use, and the study reported the temperature error between the commercial device, and the proposed patch was larger than 15%.

In this study, a semiconductor sensor-based wireless health monitoring system for measuring core body temperature is proposed. Unlike past measuring approaches, the proposed wireless temperature-measuring device is attached on the skin surface of the back under the neck, as this part of the body has thin layers of fat and muscle and so the skin temperature here is more approximate to the core body temperature. Also, the location is suitable for comfortably attaching the device to the body for longer durations. A highly accurate temperature sensor and a high-performance/low-power consumption ARM architecture microcontroller were used to develop the wireless temperature-measuring device for the skin temperature measurements. The measured data were transmitted to the receiver using a multichannel Industrial, Scientific, and Medical (ISM) band 2.4 GHz GFSK RF communication method. An algorithm was developed based on the curve-fitting method to estimate the core body temperature according to the skin temperature value. The proposed system was manufactured, and performance tests and *in vivo* experiments were conducted to confirm system performance.

## 2. Methods

**Figure 1:**
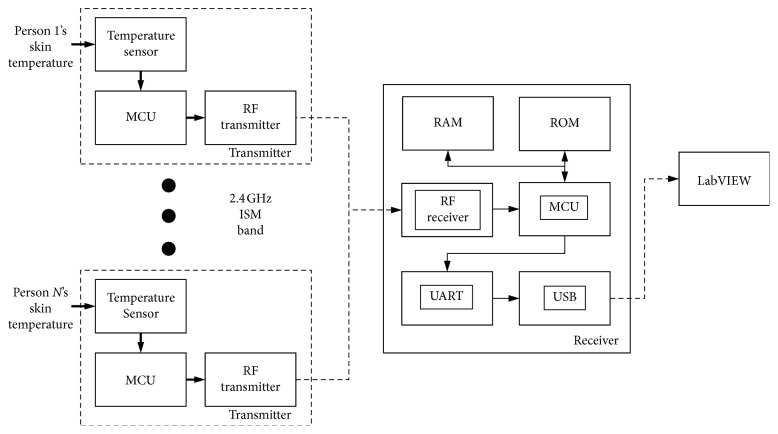
Block diagram showing the basic idea of the proposed wireless health monitoring system.

Figure 1 shows the basic idea for the proposed wireless health monitoring system composed of two parts: a wireless temperature-measuring device that is attached to the back of the body for measuring skin temperature and a receiver device for acquiring the data from the transmitter and sending the data to a computer for display and recording. In developing the wireless temperature-measuring device, a semiconductor-based microtemperature sensor, Si7021 (Silicon Labs, USA), was chosen as the sensing device for skin temperature measurement. This sensor has a small measurement error of approximately ±0.4°C at 1 Hz sampling rate in temperature measurement; this contributes to measuring skin temperature accurately with low power consumption. The EFM32WG 32-bit microprocessor (Silicon Labs, USA) was designed as the main controller for the device. This microcontroller unit (MCU) family, based on the ARM Cortex-M4 core, provides a full digital signal processing (DSP) instruction set and includes a hardware floating point unit (FPU) for faster computational performance. Also, it features up to 256 kB of flash memory, 32 kB of RAM, and CPU speeds up to 48 MHz, which are suitable to estimate the core body temperature in real time by embedding the algorithm. In addition, to minimize energy consumption, intelligent peripherals enable this MCU to control the device with high efficiency and longer battery life. In this research, the nRF24L01 (Nordic Semiconductor, Norway) transceiver was chosen to achieve wireless communication between transmitter and receiver. The transceiver is an ultralow-power 2 Mbps transceiver IC for the 2.4 GHz ISM band. The multireceiver technology that enables the receiver can communicate with a maximum of 128 transmitters simultaneously.

In designing the receiver, C8051F996 (Silicon Labs, USA), an ultralow-power MCU, was chosen to control the receiver. A highly integrated USB-to-UART bridge controller CP2102 (Silicon Labs, USA) was used inside the receiver for connecting the receiver to a computer easily. CP2102 has a simple solution for updating UART designs to USB using a minimal of components, and the printed circuit board (PCB) is an important reason to use this chip for connecting the receiver to a computer by a USB connection. The data are displayed and recorded on the computer by a developed LabVIEW program.

## 3. Experiments

### 3.1. System Manufacture

**Figure 2:**
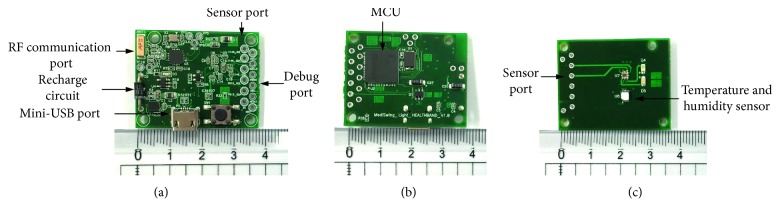
Manufactured PCBs for the wireless temperature-measuring device: (a) front view of the main board; (b) back view of the main board; (c) sensor board.

Figure 2 shows the manufactured PCBs of the proposed wireless temperature-measuring device: one is the main board (front and back views) for mounting the MCU and the wireless communication components and the other is the sensor board with temperature sensor components. The wireless temperature-measuring device had to be designed as small as possible to make it easy to attach to the back of the body. Therefore, all the PCBs were manufactured with four-layer structures, and all the components were chosen with surface-mounted device- (SMD-) type size 2012 mounted on both sides of the PCB to minimize the PCB size. A mini-USB port was fixed on the top of the PCB for connecting to the USB adapter for battery recharging. In addition, a chip shape 2.4 GHz antenna was fixed on the top of the main PCB and kept away from the MCU to prevent the electrical effect from the electronic components, which would reduce the performance of the wireless communication. The temperature sensor was designed on the other PCBs for attaching securely to the back of the body. Guide holes were designed on the same side of the two PCBs for connecting the sensor board to the main board easily. The dimensions of each PCB are 38 mm × 30 mm × 10 mm.

**Figure 3:**
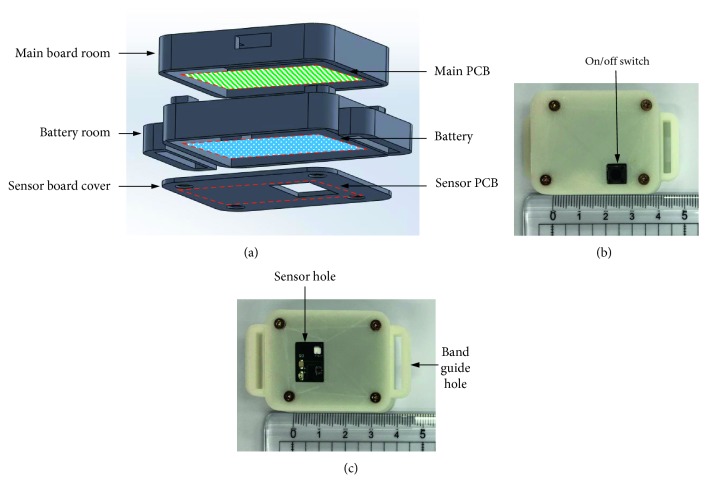
Photos of the shell for the proposed wireless temperature-measuring device: (a) 3D model of the shell structure; (b) top view of the assembled wireless temperature-measuring device; (c) bottom view of the assembled device.

Figure 3 shows the shell design in the 3D mode for the proposed wireless temperature-measuring device, and the device assembly with the manufactured shell. The shell dimensions are 40 mm × 30 mm × 17 mm and separated into two storage spaces: the upper space for attaching the main PCB and the bottom space designed as storage for a rechargeable battery and sensor PCB. The upper shell was designed with a power switch hole and USB porthole. Also, a porthole was designed on the bottom cover, which enabled the sensor to contact the skin of the back directly and completely. In addition, a 450 mAh size with 40 mm × 40 mm × 2 mm size rechargeable battery was chosen for the power supply. The shell was manufactured by 3D printer with polylactic acid (PLA) material that is known to be harmless to humans.

### 3.2. Experiments for the System Performance Test

#### 3.2.1. Temperature Measurement Performance Test for the Wireless Temperature-Measuring Device

**Figure 4:**
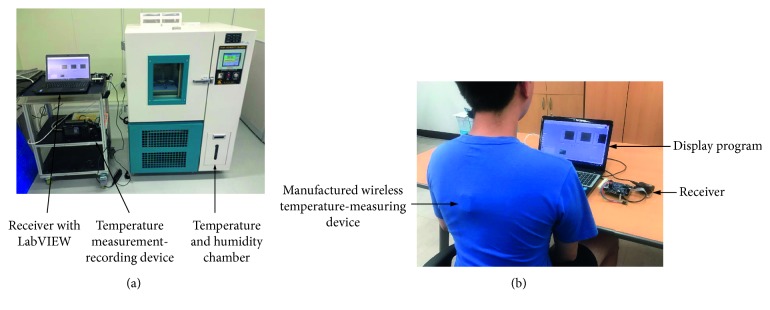
Photos of the manufactured system in the performance test: (a) temperature measurement performance test in the temperature and humidity chamber; (b) manufactured system in the *in vivo* experiment for measuring body temperature from the back of the body.

The manufactured wireless temperature-measuring device was situated in a temperature and humidity chamber (T2, YMRTC) for testing the performance of the measuring temperature as shown in Figure 4(a). The temperature in the chamber was set at an initial state of 25°C and increased by 5°C every 30 minutes, up to 40°C, a range similar to human skin temperature. Because of the temperature sensor for the chamber was at the top of the chamber, the temperature value on the chamber display was not suitable for comparing measured values from the proposed device. Therefore, a K-type thermocouple sensor was attached near the sensor hole of the manufactured wireless temperature-measuring device and connected with a midi logger GL820 (GRAPHTEC, USA) for observing temperature variation in the chamber. The data measured by the wireless temperature-measuring device were transmitted to a laptop connected to the receiver. The received temperature value was simultaneously processed and displayed with the developed LabVIEW program. In addition, a wireless communication performance test of the proposed device was conducted for observing the data rate, communication rate, and power consumption. A mixed-domain oscilloscope, MDO4104C (Tektronix, USA), was connected to the Master-In-Slave-Out (MISO) port of the MCU to monitor the wireless communication data rate. An experimental table had a receiver that connected to a laptop and could be moved far away from the wireless temperature-measuring device to find the maximum distance for wireless communication. Also, the power consumption was evaluated by a digital multimeter, Fluke 289 (Fluke, USA), connected to the power line of the wireless temperature-measuring device.

#### 3.2.2. In Vivo Experiment

Ten subjects (gender: male, age: 25 ± 1 years old) were invited to participate in the *in vivo* experiment. Before the experiment began, the subjects were required to stay in the rest state with a comfortable posture to maintain a normal body temperature [13]. As Figure 4(b) shows, the manufactured wireless temperature-measuring device was attached on the middle of the back under the neck of the subject by Micropore Surgical Tape (3M, USA). Meanwhile, an infrared thermometer, Fluke VT04 (Fluke, USA) with a measurement range from −10°C to 250°C and accuracy of ±2°C at 25°C was used to measure the temperature of the surrounding skin for comparison with the value measured by the proposed device. Ear temperature is the most popular noninvasive approach to measure the core body temperature. Therefore, ear temperature was measured by an ear thermometer (Braun, Germany) for observing the relationship between ear temperature and skin temperature. The experiment was conducted for 10 minutes, and measurement data were recorded every 30 seconds. The temperature and humidity for the experimental environment were maintained at 25°C and 50%, respectively. A consumer indoor thermometer MOG-HTC1 (B. S. Basic, Korea), with temperature measurement range from −50°C∼70°C, accuracy of 0.1°C, and the error value of ±1°C was used to monitor the environmental variation.

## 4. Results and Discussion

**Figure 5:**
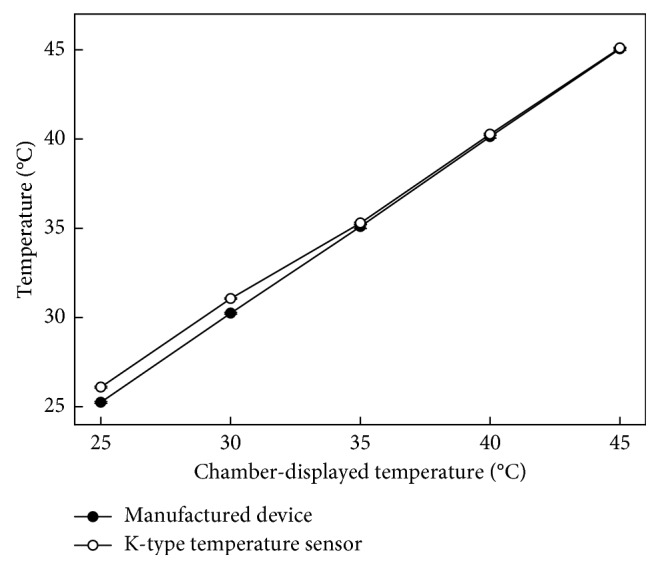
Comparison of the proposed manufactured wireless temperature-measuring device and a highly professional device in the temperature measurement performance test with a chamber temperature controlled from 25 to 45°C.

Experimental results for comparison of the manufactured wireless temperature-measuring device with the highly professional temperature-measuring device in the temperature-measurement performance test are shown in Figure 5. The temperature value measured by the proposed device was lower than the k-type sensor by 0.8°C at 25°C. The gap of the two measured values closed gradually with the increasing temperature in the chamber. At 35°C, the error value was only approximately 0.1°C. The measured temperature of the manufactured device was a little bit higher than the controlled temperature of the chamber, so it is assumed that the position of the designed sensor was close to the regulator elements of the power supply, and all the elements were packaged in the designed shell, which means the heat cannot be dissipated quickly.

**Figure 6:**
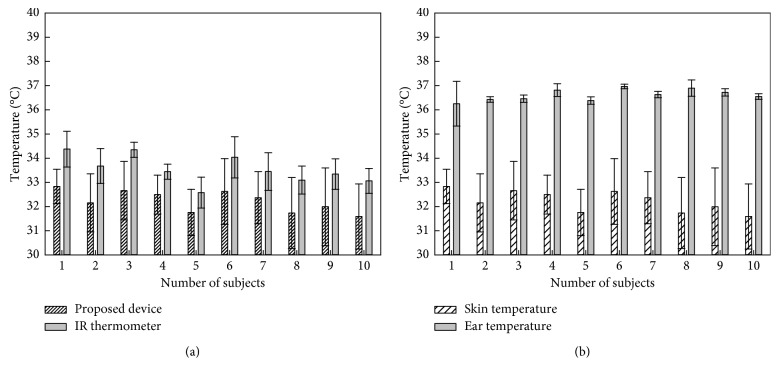
*In vivo* experimental results for observing the relationship between skin temperature and ear temperature: (a) skin temperatures were measured by the proposed device and IR thermometer for each subject; (b) comparison of the measured skin temperature and ear temperature.

Skin temperature measurement comparison results of the proposed wireless temperature-measuring device and IR thermometer for ten subjects in 10 minutes are shown in Figure 6(a). All of the subject results show that the temperature measured by the proposed device was lower than the IR thermometer by about 1°C for each subject. It was assumed that the IR thermometer has ±2°C error value and the position of the device is in front of the IR thermometer; therefore, the IR thermometer measured both the skin temperature and the proposed device's temperature. Also, as Figure 6(b) shows, the skin temperature was compared with the ear temperature measured by the proposed device and ear thermometer individually in the same condition for ten subjects. The ear temperatures for all of the subjects were close to 36.5°C, which means every subject had normothermia in these experiments. All of the experimental results show that the ear temperature was higher than the skin temperature by approximately 4°C. Other researchers such as Thomas et al., also reported these phenomena: such as a 9% variation between axillary skin temperature and rectal temperature and 16% variance between thoracic skin and rectal temperatures [14]. In this research, a 4°C error value between the back skin temperature and ear temperature means an approximately 11% variation, which is lower than the 16% variance found in comparing thoracic skin temperatures to rectal temperatures.

**Figure 7:**
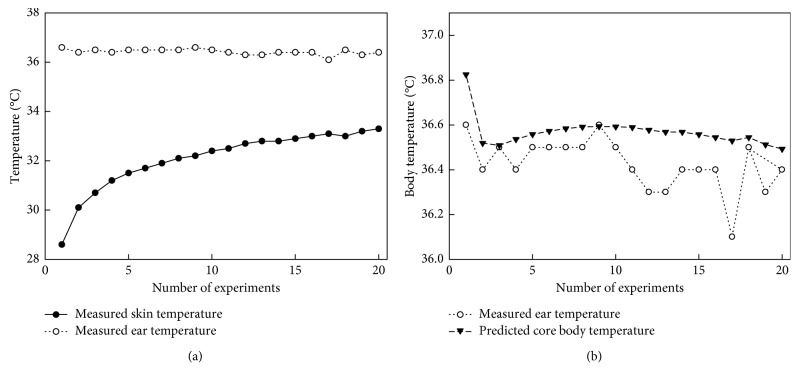
Using the developed algorithm to estimate core body temperature by skin temperature: (a) skin temperature vs. body temperature; (b) estimated body temperature vs. core body temperature.

**Figure 8:**
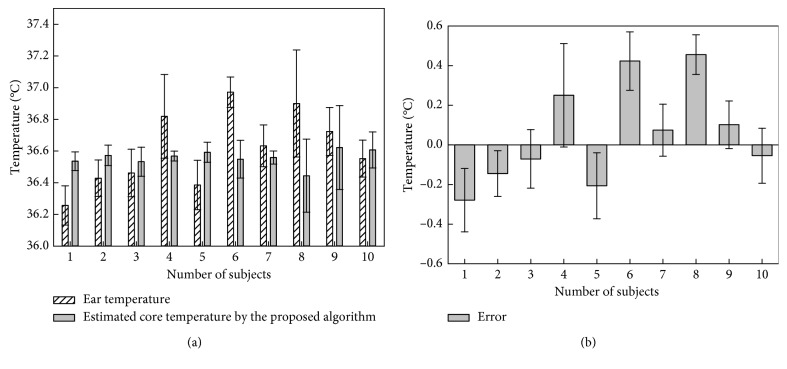
Comparison of the core body temperature and body temperature estimates for all subjects: (a) body temperature vs. estimated temperature; (b) error value between body temperature and estimated temperature.

According to the *in vivo* experimental results, an algorithm based on the curve-fitting method for estimating the core body temperature by skin temperature was designed in this research. The algorithm was found with a linear model and set as a quadratic formula with an interpolant, as equation (1) shows. Each coefficient for the equation was set with 95% confidence bounds. For evaluating the goodness of fit, the sum of squares due to error (SSE), *R*-square, adjusted *R*-square, and root mean square error (RMSE) values were observed, and the values were 33.0874, 0.0212, 0.0117, and 0.3998, respectively. An example of using the developed algorithm to estimate the core body temperature by the measured skin temperature of one subject is shown in Figure 7. As mentioned before, ear temperature is larger than the skin temperature of about 4°C in the actual measurement. Through the developed algorithm, the measured skin temperature was converted to the estimated core body temperature that closely approximates the ear temperature. In addition, the algorithm can compensate the initial value occurring when the temperature sensor is in an initial status, so the measured value is lower than the actual value. All of the experimental results processed by the developed algorithm to compare the core body temperature are shown in Figure 8. As the results show, the mean value for the error between ear temperature and estimated core body temperature is about ±0.19°C and mean bias is 0.05 ± 0.14°C; this can be explained by the accuracy of the developed algorithm, which is in the same range as the small core body temperature changes in the 10 subjects (maximum decrease of core body temperature of 0.4°C in subjects 6 and 8). However, in this paper, only the ear temperature was regarded as the reference value for the core body temperature, and the *in vivo* test was evaluated in a limited environment [16]. On the contrary, the wireless temperature-measuring device has shown a good performance when transmitting measuring data to the receiver in the operation performance test. The data transmission rate of the developed wireless communication method is about 600 kbps. And the power consumption of the wireless sensing device in operating was about 5.99 mA, and the proposed device can work without interruption about 40 hours:

(1) $$\begin{array}{c} {\text{Core}}_{\text{BodyTemp}}=0.04615\;\times \;\sin\left({\text{Skin}}_{\text{Temp}}-pi\right)-0.0006727\times \;{\left({\text{Skin}}_{\text{Temp}}-10\right)}^{2}+36.97. \end{array}$$

## 5. Conclusion

In this research, a wireless health monitoring system for measuring skin temperature from the back of the body to estimate the core body temperature was developed. The system was manufactured with a highly accurate temperature sensor, low-power consumption MCU, and multichannel ISM band RF method. According to the performance test results, the device performed well in measuring temperatures in a temperature chamber. Also, power consumption of the device during operation was approximately 5.99 mA, and the proposed device can work without interruption for approximately 40 hours. Therefore, the proposed device can be securely attached to the back of the body in order to measure skin temperature accurately for a long time. Experiment with 10 subjects in the rest status showed the measured skin temperature is lower than the ear temperature. Through the developed algorithm, this gap was compensated for, and the core body temperature that was estimated by the skin temperature approximated the ear temperature closely. However, in this paper, only the ear temperature was regarded as the reference value for the core temperature, and the *in vivo* test was evaluated in limited environment. In future work, the esophageal temperature will be considered as a gold standard for the core temperature, and some protocols such as exercise and bathing will be included for thermometer performance testing.

## Data Availability

No additional unpublished data are available.
